# Platinum-based Chemotherapy in Primary Advanced Seminoma—a Retrospective Analysis: Treatment Results at the Northern Israel Oncology Center (1989–2010)

**DOI:** 10.5041/RMMJ.10139

**Published:** 2014-01-21

**Authors:** Moshe E. Stein, Karen Drumea, Tomer Charas, Anthony Gershuny, Rahamim Ben-Yosef

**Affiliations:** 1 Northern Israel Oncology Center and Faculty of Medicine, Technion-Israel Institute of Technology, Haifa, Israel and; 2 Department of Clinical Oncology & Radiation Therapy, Queen’s Hospital, Romford, Essex, UK

**Keywords:** Cisplatin-based chemotherapy, primary advanced seminoma

## Abstract

**Objective:**

Over the past 30 years, great strides have been made in the treatment of disseminated testicular tumors. Despite the low number of patients and the rarity of studies concerning primary advanced seminoma, the efficacy of chemotherapy is clear, mainly 3–4-cisplatin-based chemotherapy. Aiming to contribute to the understanding and implementation of proper chemotherapeutic management in advanced seminoma patients, we retrospectively summarized our experience with 26 patients who were referred for platinum-based chemotherapy, post-orchiectomy to the Northern Israel Oncology Center between 1989 and 2010. Response rate, side effects, and long-term outcome were investigated.

**Methods:**

Before chemotherapy, meticulous staging was done, including tumor markers (B-human chorionic gonadotropin (B-HCG), alpha-fetoprotein (AFP), and lactic dehydrogenase (LDH)), and abdominal and pelvic computerized tomography (CT) scans were carried out.

**Results:**

All 26 treated patients achieved complete remission, clinically and symptomatically, with normalization of their CT scans. At a median follow-up of 120 months (range, 24–268 months) all patients are alive, without evidence of recurrent disease. One patient whose disease recurred twice achieved a third complete remission following salvage treatment with high-dose chemotherapy and autologous peripheral stem cell transplantation. Another patient, who preferred surveillance, relapsed abdominally after 9 months but achieved long-standing complete remission with cisplatin-based chemotherapy. Both these patients are alive with no evidence of disease. Three patients recovered uneventfully from bleomycin-induced pneumonitis.

**Conclusions:**

Advanced seminoma is a highly curable disease using platinum-based chemotherapy. Our study confirms the efficacy and safety of cisplatin-based chemotherapy in the treatment of advanced seminoma.

## INTRODUCTION

About 80% of patients with advanced seminoma (AS) (bulky adenopathy, stage IIC; or mediastinal/supraclavicular, stage IIIA) presentation can achieve a durable complete response and long-term disease-free survival following cisplatin-based chemotherapy.^1^,^2^ In recent years, cisplatin-based chemotherapy has replaced radiotherapy as the standard treatment for patients with AS. Due to the low incidence of this entity and reported studies with relatively few patients, the optimal pretreatment work-up, and especially the policy in asymptomatic post-chemotherapy residual stable masses, is unclear. Since 1989, we have treated 26 fully assessable patients with AS and report here staging methods, chemotherapy modalities, side effects, results, and survival.

## PATIENTS AND METHODS

Twenty-six patients diagnosed with AS were included in our study. All patients were referred to the Northern Israel Oncology Center following inguinal orchiectomy due to typical seminoma and the radiological diagnosis of AS, between 1989 and 2010. All patients underwent physical and neurological examinations. Complete blood count and a biochemistry profile including B-human chorionic gonadotropin (B-HCG), alpha-fetoprotein (AFP), and lactic dehydrogenase (LDH) were performed before and after initiation of chemotherapy. All patients were referred to evaluation of their disease, including abdominal and pelvic computerized tomography (CT) scans. Due to inconclusive CT results in four patients, lymphography, gallium scan, and positron emission tomography (PET) scans were done, respectively (Table 1). Response was determined by modified Memorial Sloan–Kettering Cancer Center (MSKCC) criteria.^3^ Complete response (CR) was defined as the disappearance of all clinical, radiographic, and biochemical signs, either immediately or within 1 to 4 months after completion of chemotherapy, a gradual shrinking of the abdominal mass to a stable mass less than 3 cm in size, or continuous normal tumor marker levels. Response duration and survival were measured from the end of chemotherapy.

**Table 1.** **Clinical and Pathological Characteristics.**

**Parameter**	**Value**
**Age (mean; range), years**	39.5; 17–66
**Origin**	
European Jews	18
Non-European Jews	3
Christian Arabs	3
Moslem Arabs	2
**Place of birth**	
Europe	3
Israel	21
USA	1
Russia	1
**Accompanying local conditions**	
Hernioplasty	1
Cryptorchidism	4
Hydrocele	2
Cryptorchidism, bilateral	2
**Disease sites**	
Left testicle	11
Right testicle	14
Bilateral	1
**Presenting symptoms**	
Testicular enlargement/swelling	13
Palpable mass	16
Testicular pain	4
Abdominal/pelvic pain	7
Supraclavicular palpable mass	1
**Duration of symptoms (mean; range), months ^a^**	1.4; 1–12
**Radiological measures**	
Testicular ultrasound	26
IVP	1
Lymphography	1
CT	26
Abdominal ultrasound	5
PET-CT	6
**Elevated tumor markers**	
B-Human chorionic gonadotropin	8
Lactic dehydrogenase	12
**Staging**	
IIB	7
IIC	16
IIIA	3
**Testicular pathology (post-orchiectomy)**	
Pure (classical) seminoma	26 ^b^
***Invasion of:***	
Tunica vaginalis	5
Lympho-vascular spaces	5
Spermatic cord	2
Rete testis	2
Epididymis	2
IGCN	4
**Testicular seminoma pathological staging**	
T1	21
T2	3 ^c^
T3	2 ^d^

**Notes**

a Three patients presented with symptom duration of 1, 2, and 4 years.

b The abdominal masses developed in the majority of patients simultaneously with the testicular seminoma.

c All T2 patients demonstrated invasion of the tunica vaginalis or epididymis with lympho-vascular invasion.

d The T3 patients exhibited spermatic cord invasion.

IVP, intravenous pyelography; CT, whole-body computerized tomography scans; PET-CT, positron emission tomography scans; IGCN, intratubular germ cell neoplasm.

## RESULTS

Patient characteristics are shown in Table 1. Mean age of patients was 39.5 years (range, 17–66 years), and 18 out of 26 (69%) patients were Jews of European descent, born in Israel. Six patients had a previous history of cryptorchidism, and the relation between the right and left testicle was 15:12. One patient presented with bilateral testicular tumors. The main presenting symptoms were painless testicular enlargement, swelling, and a palpable mass within the affected testicular sac. In seven patients, abdominal and/or pelvic pain appeared simultaneously (Table 1). The mean duration of symptoms before consulting a physician was 1.4 months. Three patients ignored the symptoms for 1, 2, and 4 years, respectively, a fact which did not affect their response and survival chances.

Tumor markers were performed in all patients (Table 1). AFP was negative in all patients. Elevated levels of LDH and B-HCG were measured following every cycle and decreased gradually after orchiectomy, normalizing upon entering complete remission. No further elevation was observed during follow-up. All 26 patients demonstrated typical (classical) seminoma (Table 1) with various degrees of invasion and involvement of anatomic structures of the testis. Twenty-one (90%) patients were considered to have pathological pT1 disease. Only three patients had lympho-vascular invasion, and none of them had perineural involvement. Two patients showed spermatic cord invasion (pT3), but T-classification was not a prognostic factor in response assessment. Intratubular germ cell neoplasm (IGCN) was found in four patients.

All radiological measures, mainly CT scan, exhibited retroperitoneal and/or pelvic lymphadenopathy. Unilateral hydronephrosis was seen in six patients. The Royal Marsden Staging Classification,^4^ as seen in Table 1 (IIB, 2–5 cm; IIC, more than 5 cm or very bulky disease; IIIA, mediastinal/supraclavicular lymphadenopathy), was implemented. In a few patients, the bulky masses exceeded 9 cm and caused hydronephrosis with moderate to severe abdominal pain.

All patients were treated with cisplatin-based chemotherapy, mainly bleomycin/cisplatinum/etoposide (BEP) combination,^4^ for 3–4 cycles (Table 2). All patients achieved immediate/rapid or slow complete regression as demonstrated by normalization of previously elevated LDH and B-HCG levels and by CT scans. In five (19%) patients, in whom the tumoral mass shrinkage was very slow, follow-up consisted of CT scans, and six patients also had PET scans (Table 2). In two patients, para-aortic lymph node packets could be followed on CT scans during 1 year of follow-up until disappearance. No evidence of persistent or regrowing masses was demonstrated. The three patients with pathologically and radiologically confirmed IIIA disease also responded completely to BEP. After a median follow-up of 120 months (range 24–268 months) all patients are alive with no evidence of disease.

**Table 2.** **Treatment Modalities, Side Effects, and Results.**

	**No. of Pts.**
**Chemotherapy schedule** ^a^	
Cisplatinum/bleomycin/etoposide (BEP)	22
Carboplatin/etoposide/bleomycin	2
Carboplatin/etoposide	1
Cisplatinum/etoposide (EP)	6
**Side effects**	
Neutropenic fever	4
Mild peripheral neuropathy	2
Bleomycin lung toxicity	3
Mucositis, grade II	2
Temporary partial hearing loss	1
Tinnitus	1
Herpes zoster	1
**Response rate**	
Complete remission	26
**Assessment of response**	
CT	20
PET-CT	6
Gallium scan	2

a Four patients received more than one schedule.

One patient (Table 3, #22) developed lung metastases 4 years after his first CR. He responded to vinblastine/ifosfamide/cisplatinum (VeIP) salvage chemotherapy for 4 years, but eventually his disease recurred in the lungs and pelvis. This patient entered a third CR following high-dose chemotherapy (HDCT) with autologous peripheral stem cell transplantation (APSCT) and local radiation therapy, resulting in long-term (third) CR. Currently, 16 years following his last treatment, he is alive with no evidence of testicular tumor. Another patient (#19) preferred surveillance initially, but relapsed after 9 months with a IIC abdominal mass and achieved prompt and durable complete remission with three BEP cycles.

**Table 3.** **Staging, Chemotherapy Regimens, Response, and Latest Status.** All studied patients were contacted by the departmental secretary on January 1, 2012 and were found to be alive with no evidence of their previous testicular tumor. Remission was assessed and confirmed clinically, radiologically, and biochemically (B-HCG, LDH).

**Pt. No. (Age at Diagnosis)**	**Side of Disease**	**Pathologic Staging of Testicular Seminoma**	**Stage**	**Regimen × No. of Cycles**	**Side Effects**	**Response**
#1 (60)	Left	T2	IIC	BEP × 4	Neutropenic fever Mild peripheral neuropathy	CR
#2 (17)	Left	T1	IIC	BEP × 3	–	CR
#3 (39)	Right	T2	IIC ^a^	BEP × 3	–	CR
#4 (47)	Left	T1	IIC ^b^	BEP × 3; EP × 1	Bleomycin lung toxicity ^c^ Mild peripheral neuropathy	CR
#5 (55)	Left	T1	IIC ^d^	BEP × 1; Carboplatin/etoposide/bleomycin × 2; Carboplatin/etoposide × 1		CR ^e^
#6 (36)	Left	T1	IIC	BEP × 3	Bleomycin lung toxicity ^f^	CR
#7 (42)	Right	T1	IIIA ^g^	BEP × 3	Mucositis grade II	CR
#8 (43)	Right	T2	IIC	Carboplatin/bleomycin/etoposide × 4		CR
#9 (41)	Right	T1	IIC	EP × 3	Neutropenic fever	CR
#10 (33)	Left	T1	IIC	BEP × 3		CR
#11 (50)	Left	T1	IIC	CDDP/VP-16 × 4	Neutropenic fever	CR
#12 (53)	Bilateral	T1	IIC	BEP × 4	Mild hearing loss	CR
#13 (23)	Right	T1	IIB	BEP × 3; EP × 1		CR
#14 (66)	Right	T1	IIIA	BEP × 3; EP × 1		CR
#15 (32)	Right	T2 ^h^	IIC	BEP × 3; EP × 1		CR
#16 (46)	Right	T2	IIC ^i^	BEP × 3	Mucositis grade III	CR
#17 (42)	Right	T1	IIB	BEP × 3	Mild tinnitus	CR
#18 (27)	Left	T1	IIIB	BEP × 3		CR
#19 (29)	Right	T1 ^h^	IIC	BEP × 3		CR ^j^
#20 (34)	Left	T2 ^j^	IIB	BEP × 3	Bleomycin lung toxicity ^k^	CR
#21 (32)	Right	T1	IIB	BEP × 3		CR
#22 (21)	Right	T1	IIC	BEP × 4		CR ^l^
#23 (31)	Right	T1	IIB	BEP × 3	Herpes zoster	CR ^m^
#24 (43)	Left	T1	IIC	BEP × 3		CR ^n^
#25 (51)	Left	T1	IIIA ^o^	BEP × 3		CR
#26 (33)	Right	T1	IIB	BEP × 4	Neutropenic fever	CR

a Patient #3 presented with very advanced retroperitoneal mass, measuring 10 × 9 × 13 cm; unilateral hydronephrosis; involved right-sided ureter.

b Patient #4 presented with massive retroperitoneal and pelvic lymphadenopathy, hydronephrosis and hydroureter.

c Bleomycin lung toxicity (BIP) = clinically asymptomatic, radiological diagnosis; cumulative bleomycin dose 180 units.

d Retroperitoneal lymphadenopathy, measuring 9.5 × 13.5 × 7.5 cm; left-sided hydronephrosis; involved diaphragmatic crus.

e Slow radiological response—it took several months until complete resolution of residual mass.

f Clinically asymptomatic, radiological manifestation; reaching total bleomycin dose 240 units.

g IIIA = retroperitoneal, mediastinal, and left-sided supraclavicular groove lymphadenopathy; pathologically confirmed.

h Seminoma with intratubular germ cell tumor.

i Huge retroperitoneal mass, obstructed left kidney with resulting hydronephrosis, necrotic mass in the midline, inguinal and external iliac lymphadenopathy.

j Patient preferred surveillance initially; relapsed 9 months later with abdominal pain and retroperitoneal mass (52 mm); in complete remission.

k Very symptomatic with radiological signs; treated successfully with high-dose steroids; cumulative bleomycin dose 240 units.

l Patient relapsed with lung metastasis 1 year following BEP; responded to second-line chemotherapy with ifosfamide-based chemotherapy for 4 years; disease recurred in lungs and pelvis; entered third CR following HDCT with APSCT and local radiation therapy; alive NED for 168 months.

m PET-CT-confirmed CR.

n Gallium scan-proven CR.

o Biopsy-proven metastatic lymphadenopathy in the left supraclavicular groove.

CR, complete response; EP, etoposide/cisplatinum.

**Figure 1.**
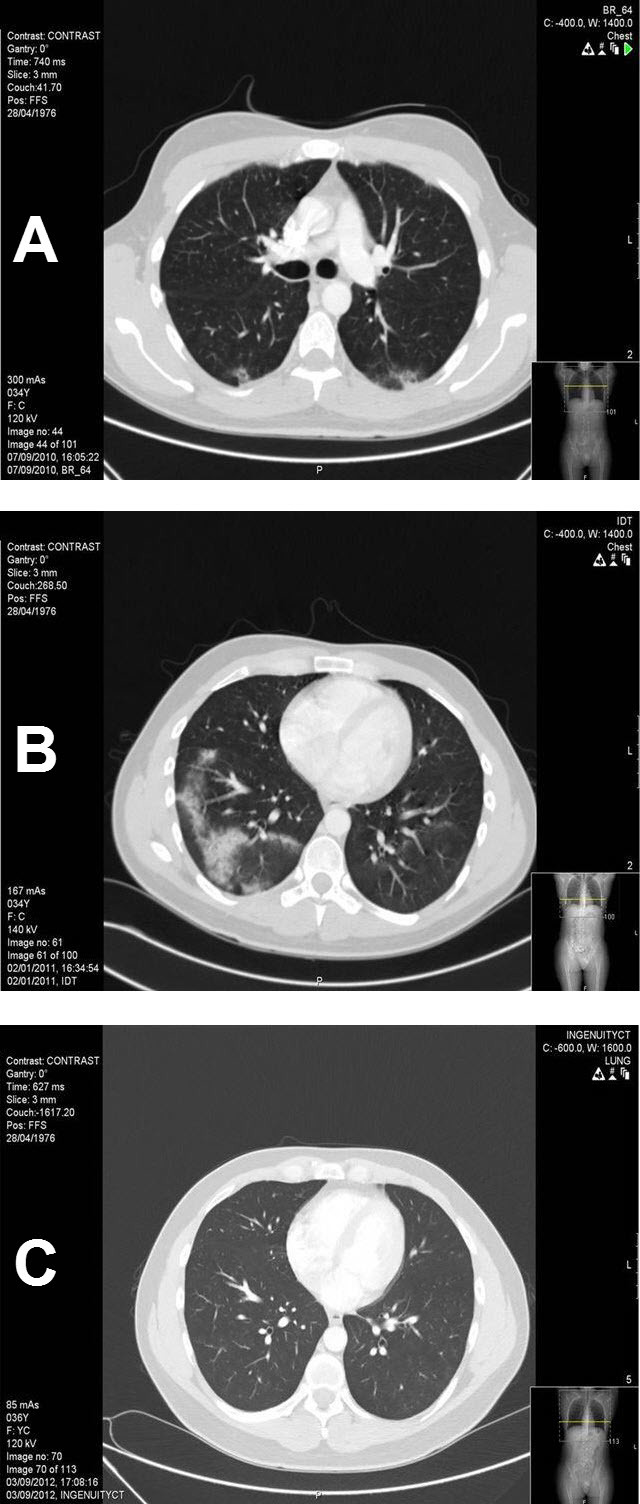
**A CT Scan Following Completion of BEP Regimen (cumulative bleomycin dose 240 units) at 2 Months (A), 5 Months (B), 10 Months (C).** **A)** 2 months: Interstitial alveolar infiltrates in the posterior, hanging parts of the right and left lower lobes; **B)** 5 months: Alveolar shadows in various sizes confined to both lung bases, more on the right side but also on the right middle and upper lobes—clear worsening compared to 1A; **C)** 10 months: Clear regression and improvement in the right lower and right middle lobes.

Side effects were manageable (Table 2). In three patients, cisplatinum was replaced by carboplatin due to the development of tinnitus and mild hearing loss, respectively. In seven patients, bleomycin was omitted for the fourth cycle, and the fourth cycle was modified in two patients due to neutropenic fever. Within a range of 2–4 months, three patients developed clinical and radiological signs of bleomycin lung toxicity after reaching a cumulative dose in the range of 180–240 units of bleomycin. Clinically, they presented with non-productive cough, exertional dyspnea, and low-grade fever. The chest X-ray showed bilateral, bibasilar infiltrates, with later consolidation (Figure 1, panels A and B) which was totally reabsorbed with no progression (Figure 1, Panel C) into irreversible diffuse fibrosis. The three patients responded well to high-dose steroids and broad-spectrum antibiotics.

Following a thorough search on the website of the Ministry of Interior Affairs, we found that all the treated patients are alive and well with no evidence of their previous testicular tumor. Unfortunately, due to the lack of a data base, we were unable to evaluate long-term severe side effects, such as second primaries, solid or hematological malignancies. We plan it for an on-coming study.

## DISCUSSION

Our long-term results, comparing to other studies,^4^,^5^ show that AS is highly sensitive to platinum-based chemotherapy, with up to 85%–100% of patients showing durable complete remission^1^,^5^ and survival rates greater than 90% in 10 years.^1^,^6^ Toxicity was mild and manageable in most of the studies, apart from reports of late second primaries, either solid or hematological in nature^7^–^9^ which can be a real challenge in cured patients.

Most common acute and chronic side effects of BEP or other platinum-based chemotherapy consist of myelosuppression of various grades, nephrotoxicity, peripheral neuropathy, cardiovascular diseases and ototoxicity. Only four of our patients developed neutropenic fever after three cycles, recovering uneventfully, and two other patients developed reversible mild peripheral neuropathy. Hypomagnesemia and reduced glomerular filtration rate were found in patient #22 following ifosfamide-based salvage regimen.

Bleomycin is an important component of the BEP regimen, usually given in 30 units intravenously weekly (days 2, 9, and 16) in three to four cycles, up to a cumulative dose of 270–360 units. Such cumulative doses might cause the feared and sometimes fatal bleomycin-induced pneumonitis (BIP). Three of the patients (Table 3, patients #4, #6, and #20) developed BIP. All three patients exhibited the classic clinical signs, such as severe non-productive cough, exertional dyspnea and fever, and the characteristic radiological signs of bilateral bi-basilar infiltrates progressing into airspace consolidation and ground-glass opacities. All three patients responded promptly to high-dose steroids and broad-spectrum antibiotics. Generally, BIP may occur in up to 46% of patients treated with bleomycin-containing regimens, with mortality up to 3%.^10^ A central pathological event in the pathogenesis of BIP is endothelial damage of the lung vasculature, mediated through cytokines and free radicals which contribute to endothelial cell damage and to subsequent infiltration of inflammatory cells into the interstitium, activation of fibroblasts and accompanying excess collagen deposition, and the irreversible process of fibrosis continuing until respiratory failure.^10^,^11^ Diagnosis is based on clinical symptoms, radiography, and pulmonary function tests. In unclear cases bronchoscopy with broncho-alveolar lavage and/or lung biopsy should be done. Un- or undertreated BIP progresses to severe dyspnea at rest, tachypnea, and cyanosis, and radiologically into diffuse and massive interstitial/alveolar infiltrates, lobar consolidation, and diffuse end-stage fibrosis.^10^–^13^ Under generally recognized risk factors of BIP, we include age above 40, administration route (continuous infusion versus bolus; intramuscular versus intravenous), smoking, decreased renal function tests, previous radiation therapy to the chest, high inspired oxygen, concomitant use of other chemotherapeutic agents (platinum analogues), granulocyte-colony-stimulating factors and cumulative dose of bleomycin.^10^ O’Sullivan et al.^14^ concluded that the three main factors predicting the highest probability of BIP are a glomerular filtration rate of less than 80 mL/min, cumulative doses higher than 300 units, and age over 40. Therefore, some authors^10^,^15^ recommend lowering the dose from 360 to 270 units and even lower, but not omitting this agent. Continuous radiological and lung function tests during and after chemotherapy are recommended. There is no effective treatment of BIP, although steroids are widely applied successfully, with or without antibiotics. Experimental agents aiming at regression of BIP which also proved clinical efficiency are pentoxifylline, imatinib as a novel anti-fibrotic agent, and bleomycin hydrolase.^16^–^18^

In the long-term follow-up of AS patients treated with platinum-based chemotherapy, physicians should be on the alert for late cardiovascular events, renal dysfunction hypercholesterolemia, weight gain, erectile dysfunction, and high blood pressure. Due to cumulative etoposide doses of 2,000 mg/m^2^, equal to four cycles of BEP, a 4.7% cumulative risk of leukemic complications was seen. It appeared 5.7 years after the etoposide-containing chemotherapy.^7^,^8^

One of our patients (Table 2, #19) relapsed in the lungs 1 year following CR on BEP. He responded completely to the VeIP second-line chemotherapy and showed no evidence of disease for 4 years. Disease recurred in the lungs and pelvis, and he entered a third and long-term CR with high-dose chemotherapy plus autologous stem cell support and local radiation therapy. Miller et al.^19^ demonstrated the efficacy of VeIP in recurrent seminoma; 83% of his patients achieved complete remission, and one patient was rendered disease-free following resection of residual carcinoma. Side effects were manageable apart from hematological toxicity which necessitated the regular use of growth factors. Fifty-four percent of the patients are long-term survivors.

An important approach in refractory AS might be high-dose chemotherapy, albeit with major toxicities. As part of phase I/II studies, Rick et al.^20^ used conventional chemotherapy prior to HDCT in refractory or relapsed seminoma; 33% of their patients became disease-free, and 5/13 (38%) were alive at a median follow-up of 4.5 years. Agarwala et al.^21^ confirmed high rates of both CR and overall survival with salvage high-dose carboplatin/etoposide with peripheral blood stem cell transplantation. Despite three therapy-related deaths, two due to acute myelogenous leukemia, they proved better cure rates with HDCT in first relapse over ifosfamide/cisplatin-based conventional chemotherapy. From these and other studies we can adopt the suggestions of Rick et al.^20^ that firm conclusions are still limited by the small number of patients and the prospective nature. Analyzing their studies, Rick et al.^20^ and Agarwala et al.^21^ suggest that seminoma patients with adverse prognostic factors, such as non-pulmonary visceral metastases, short relapse-free survival, and cisplatin-refractory tumors, had less benefit from HDCT. Therefore, Lorch et al.^22^ developed an international prognostic factors model for germ cell tumor patients who experience treatment failure with cisplatin first-line chemotherapy which might help optimizing the treatment decision in those patients. In another prospective study,^23^ patients achieved durable long-term survival after single as well as sequential HDCT, albeit with some toxicity-related deaths. We can conclude that patients with an incomplete response to first-line treatment and those with short relapse-free intervals might profit from early treatment intensification. However, further long-term, prospective studies with large cohorts of patients are needed to evaluate the role of HDCT in refractory/relapsing AS.

Four of our patients demonstrated IGCN in their primary testicular seminoma pathology. IGCN or carcinoma *in situ* (CIS) preceded the development of seminoma in adults^24^ but does not have any effect on prognosis. Morphologically, CIS cells resemble seminoma cells, and cytologically there is no difference between the CIS cells that transform into seminomas and those that develop into non-seminomas. The incidence of IGCN is less than 0.3% in the general population but somewhat higher (0.5%) in patients with cryptorchid testes,^25^ such as three of our patients with IGCN who were cryptorchid.

Staging of AS (IIB versus IIC versus IIIA) is important for tailoring appropriate treatment with minimal side effects. Anatomical staging techniques, such as CT scan, intravenous pyelography (IVP), ultrasound, and lymphangiography, have severe limitations in identifying the exact extension of the lymphadenopathy, with reported false-negative rates of 59% for CT scan and 64% for lymphangiography.^26^ Enlarged lymph nodes on CT or filling defects on lymphography are not absolutely reliable for the diagnosis of AS, and lymphography is not used anymore in the staging of AS. Gallium scan has also been used in AS^27^ but was not shown to be beneficial in differentiating necrotic tissue from viable seminoma and is currently outdated.

Hain et al.^26^ and Cremerius et al.^27^ suggest PET-CT techniques for the initial staging of AS and follow-up of post-chemotherapy residual mass. In its ability to assess metabolic function of tissue through assessing the rate and quantity of tumor uptake of the glucose analogue 2-fluoro-2-deoxyglucose (FDG), PET-CT is a function which can reliably predict the presence or absence of viable tumorous tissue. There were also worrying numbers of false-positive PET results in the initial work-up of seminoma. Hence, the exact role of PET-CT in the initial staging of seminoma should be defined by large, prospective studies.

Five (23%) of our patients demonstrated slow regression of chemotherapy-treated abdominal masses. Hence, these patients were followed with repeat CT scans and tumor markers, and two also had PET scans. After a median follow-up of 4 months, all showed normalization of tumor markers, three out of five with total regression of lymphadenopathy. In two patients, para-aortic lymph node packets less than 3 cm in size with stable appearance could be exhibited until 1 year after completion of chemotherapy.

It is well known that up to 80% of patients with AS are found to have radiographically detectable residual post-chemotherapy masses,^4^,^28^ and there is still controversy about the accurate management of the asymptomatic, marker-negative mass. Surgery was suggested as an option in selected patients with a discrete mass over 3 cm or if there is evidence of local disease progression. On the other hand, opponents of the surgical approach^29^,^30^ suggest that, unlike non-seminomatous germ cell tumors, there is no option for diagnosing mature or immature teratoma in resected specimens, and the incidence of viable tumor is between 0% and 15% in residual masses, which are very sensitive to radiation therapy or salvage chemotherapy. Mosharafa et al.^29^ and others^30^,^31^ suggested that platinum-based chemotherapy in AS induces a dense desmoplastic reaction resembling retroperitoneal fibrosis that encases major vascular structures which might necessitate additional intraoperative procedures or vascular reconstruction. Seminomatous elements in patients undergoing post-chemotherapy retroperitoneal lymph node dissection were associated with a higher rate of intraoperative procedures and postoperative complications compared to patients without seminomatous elements. Friedmann et al.^6^ and Fossa et al.^32^ also concluded that surgical resection in seminoma patients is associated with excessive surgical morbidity. Other prognostic factors for intraoperative morbidity besides the seminoma histology were para-caval location of residual mass and radiologically poorly defined post-chemotherapy masses which mostly proved to be solely fibrosis and/or necrosis. The policy of the Indiana University Group^33^ is to observe patients with stable post-chemotherapy masses. The SIU/ICUD Consensus Meeting on Germ Cell Tumors suggests that even residual masses larger than 3 cm in diameter should be referred to close observation with all radiological tools.^34^ As an exception, Ravi et al.^35^ proposed the addition of intraoperative radiation (20 Gy) following resection of masses over 3 cm, but the general consideration is against radiation therapy because about 70% of patients might be unnecessarily exposed to radiation and to the risks of long-term side effects, including bone marrow and radiation-induced second primaries. Duchesne et al.^36^ found a progression-free survival of 88% uninfluenced by additional post-chemotherapy radiation.

The SEMPET trial^37^ was conducted in 19 oncology centers and summarized 177 PET-CT results in post-cisplatin-based chemotherapy for seminoma residual lesions which were correlated with either histology of the resected lesion or the clinical outcome. This trial gave a clear confirmation of the high specificity and sensitivity of PET-CT for evaluating post-chemotherapy seminoma residuals. They concluded that PET scan remains a valuable tool for clinical decision-making and spares unnecessary therapy. The 2010 major review by Rioja et al.^38^ came to the conclusion that PET is the best predictor of viable residual tumor in post-chemotherapy residual masses and should be used as a standard tool for clinical decision-making. These results were reproduced by Becherer et al.,^39^ corroborating that PET contributes to the management of residual seminoma, especially in terms of avoiding unnecessary surgery. In AS and post-chemotherapy residual mass less than 3 cm, [F-18]FDG is able to differentiate between non-viable and viable lesions, thus assigning PET-negative patients to a lower-risk group in which surveillance is justified. A protocol of active surveillance for patients with residual post-chemotherapy masses from AS, regardless of size, combining clinical and biochemical findings and CT and PET scans, has been employed at the University of California.^30^

## CONCLUSION

In conclusion, AS is very responsive to cisplatin-based and high-dose chemotherapy. Regular CT scan is an important tool in the initial staging and follow-up. Residual post-chemotherapy masses with negative PET scan and normal markers should be part of the surveillance policy, aiming to diagnose recurrent disease or second primaries.

## Abbreviations:

AFP: alpha-fetoprotein;
APSCT: autologous peripheral stem cell transplantation;
AS: advanced seminoma;
BEP: bleomycin/cisplatinum/etoposide;
B-HCG: B-human chorionic gonadotropin;
BIP: bleomycin-induced pneumonitis;
CIS: carcinoma *in situ*;
CR: complete response;
CT: computerized tomography;
FDG: 2-fluoro-2-deoxyglucose analogue;
HDCT: high-dose chemotherapy;
IGCN: intratubular germ cell neoplasm;
IVP: intravenous pyelography;
LDH: lactic dehydrogenase;
MSKCC: Memorial Sloan–Kettering Cancer Center;
PET: positron emission tomography;
SEMPET: Seminoma and PET-CT Trial;
SIU/ICUD: Societé Internationale d’Urologie/International Consultation on Urological Disease;
VeIP: vinblastine/ifosfamide/cisplatinum.
